# Rapid Progression of a CT-Defined Vulnerable Coronary Plaque: A Multimodality Imaging Case Report

**DOI:** 10.1155/cric/9999700

**Published:** 2025-10-16

**Authors:** B. S. H. Hagen, P. A. Diemen, M. J. Bom, S. P. Schumacher, A. C. van Rossum, A. Nap, M. P. Opolski, C. J. W. Verouden, I. Danad, P. Knaapen

**Affiliations:** ^1^Department of Cardiology, Amsterdam UMC, Vrije Universiteit Amsterdam, Amsterdam, the Netherlands; ^2^Department of Interventional Cardiology and Angiology, National Institute of Cardiology, Warsaw, Poland

**Keywords:** [^15^O]H_2_O positron emission tomography perfusion imaging, coronary computed tomography angiography, intraplaque hemorrhage, percutaneous coronary intervention, rapid plaque progression, vulnerable plaque

## Abstract

Rapid progression of vulnerable plaques due to intraplaque neovascularization and hemorrhage is deemed as an important process preceding plaque rupture leading to myocardial infarction. We present a patient with a rapidly progressive vulnerable plaque assessed by means of coronary computed tomography angiography and serial invasive coronary angiography.

**Summary**

• It is important to be aware of rapid plaque progression among patients with a CT-defined vulnerable plaque and progressive symptoms.
• Ongoing trials may help expand our treatment options in patients with a vulnerable plaque, potentially preventing adverse clinical outcomes.

## 1. Introduction

The present case report involves a patient with rapid progression of a coronary computed tomography angiography (CCTA)–derived vulnerable plaque leading to debilitating symptoms. This case report demonstrates one of the pathophysiological mechanisms underlying rapidly progressive angina pectoris.

## 2. Case Report

### 2.1. First Presentation

#### 2.1.1. History of Presentation

An 80-year-old male, with a family history of cardiovascular events, presented at the outpatient clinic with new-onset exercise-induced angina pectoris and dyspnea for 3 months. His physical examination was unremarkable. The cardiovascular risk factors and medication at the initial presentation are presented in Table 1.

#### 2.1.2. Past Medical History

Three years prior to presentation: Presentation at the emergency department with paroxysmal regular narrow complex tachycardia, with the following differential diagnoses: atrioventricular nodal reentrant tachycardia (AVNRT), atrioventricular reentrant tachycardia (AVRT), or atrial tachycardia.

One year prior to presentation: Echocardiography: normal left and right ventricular function and dimensions, normal atria, mild aortic valve insufficiency, and no significant valvular abnormalities.

#### 2.1.3. Working Diagnosis

The diagnosis was angina pectoris due to obstructive coronary artery disease (CAD).

#### 2.1.4. Investigations

**Figure 1:**
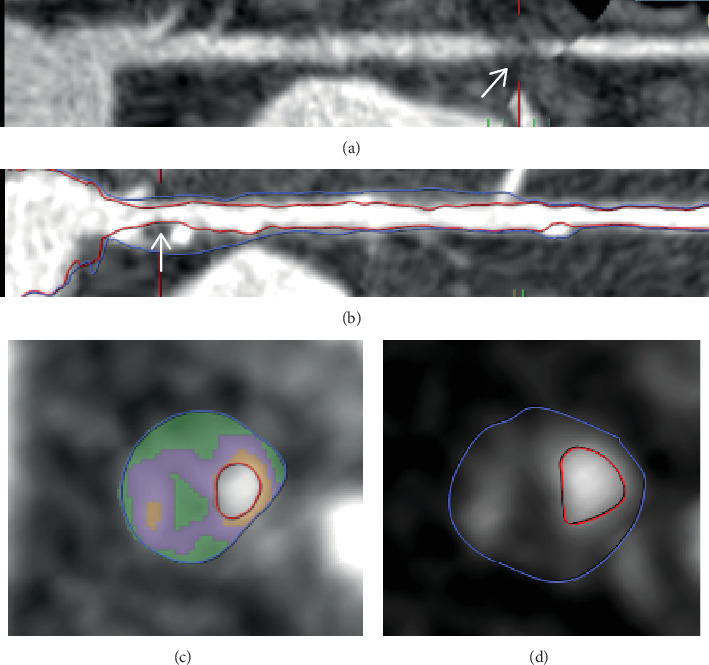
Coronary computed tomography angiography. Coronary computed tomography angiography demonstrated a severely stenotic noncalcified plaque in the OM branch (arrow) (a). The LAD was diffusely diseased with a plaque of moderate severity in the proximal part of the artery, marked by the arrow (b). Plaque analysis of the proximal LAD lesion revealed a vulnerable plaque based on adverse plaque characteristics: spotty calcification (orange), low attenuation (green), and positive remodeling (b–d). Abbreviations: LAD: left anterior descending artery, OM: obtuse marginal.

The patient's resting electrocardiogram (ECG) was normal and he subsequently underwent CCTA. The right coronary artery (RCA) had mild irregularities. Regarding the left coronary system, the obtuse marginal (OM) had an obstructive noncalcified plaque that led to significant lumen narrowing (Figure 1a). Furthermore, the proximal left anterior descending artery (LAD) harbored a plaque of moderate severity (Figures 1b, 1c, and 1d). In-depth analysis of this proximal LAD plaque demonstrated an area of low attenuation, defined by < 30 Hounsfield units, indicative of a lipid core within the plaque. Furthermore, the region with high attenuation indicated the presence of fibrous tissue and spotty calcification. Lastly, the plaque demonstrated outward remodeling. Considering these plaque characteristics, the lesion was deemed a vulnerable plaque [1].

#### 2.1.5. Management

**Figure 2:**
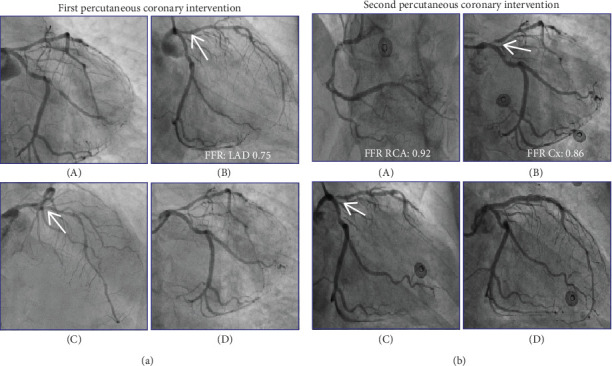
Invasive coronary angiography. (a) The first ICA revealed a (A) subtotal occlusion in the OM branch. The LAD (arrows) was diffusely diseased, functional interrogation demonstrated a gray-zone FFR of 0.75 (B, C). The OM was successfully treated by PCI (D). (b) The second ICA showed stenoses in the (A) RCA and (B) Cx were of intermediate severity with FFR values above the threshold for revascularization (0.92 and 0.86, respectively). The vulnerable plaque in the proximal LAD (arrows) had progressed to a subtotal stenoses (B, C). The LAD was successfully treated by PCI (D). Abbreviations: Cx: circumflex artery, FFR: fractional flow reserve, ICA: invasive coronary angiography, PCI: percutaneous coronary intervention, RCA: right coronary artery, other abbreviations as in Figure 1.

The patient was prescribed an aspirin, a short- and long-acting working nitrate, a *β*-blocker, and a statin (Table 2). One month after CCTA, the patient underwent an invasive coronary angiography (ICA) that revealed a subtotal occlusion of the OM (Figure 2a (A)). The LAD was diffusely diseased and had a proximal stenosis of approximately 50% (Figure 2a (B,C)). Functional interrogation of the LAD revealed a gray-zone fractional flow reserve (FFR) of 0.75. An instantaneous wave-free ratio (iFR) pullback demonstrated a gradual decline in values from the ostium to the midsegment of the proximal LAD, without any distinct focal step-up. After careful consideration, the operator decided to defer percutaneous coronary intervention (PCI) of the LAD given the diffuse disease and subtotal occlusion of the OM which was deemed a likely cause of the patient's symptoms. Subsequently, a PCI of the OM was performed (Figure 2a (D)). Due to the tortuous anatomy of the target vessel, careful manipulation of the equipment was required. A drug-eluting stent (DES) with a diameter of 2.75 mm was deployed in the mid-OM segment with an excellent angiographic outcome, with good stent apposition and preserved flow. The LAD was treated conservatively given the diffuse disease and gray-zone FFR. After PCI, clopidogrel 75 mg once daily was prescribed for 1 year.

#### 2.1.6. Follow-Up

Following PCI, the patient remained symptom-free and underwent an exercise stress test, which showed no signs of ischemia.

### 2.2. Second Presentation

#### 2.2.1. History of Presentation

The patient had been free of symptoms for 1 month, after which exercise-induced angina (similar to the first presentation) reoccurred. Over the following months, symptoms progressively worsened, occurred at lower levels of exertion, and lasted longer before resolving.

#### 2.2.2. Working Diagnosis

Diagnosis was progressive angina pectoris due to obstructive CAD.

#### 2.2.3. Investigations

**Figure 3:**
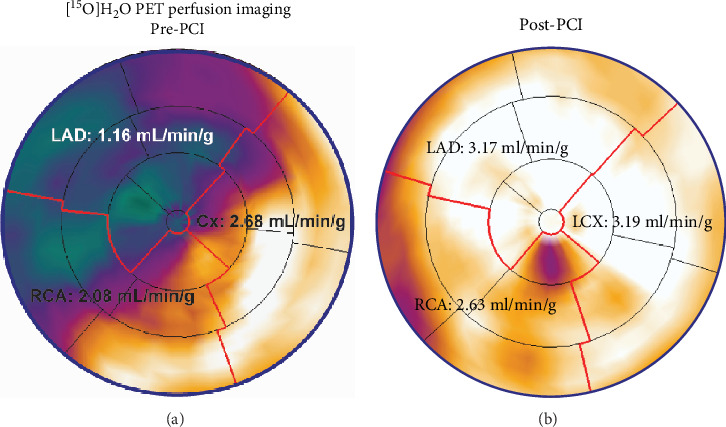
[^15^O]H_2_O PET perfusion imaging. The PET before the second PCI (a) imaging demonstrated a large perfusion defect in the LAD territory with a hyperemic MBF below the ischemic threshold (≤ 2.3 mL/min/g). The PET imaging after the second PCI (b) revealed normal hyperemic MBF in all vascular territories. Abbreviations: MBF: myocardial blood flow, PET: positron emission tomography, other abbreviations as in Figures 1 and 2.

The patient underwent quantitative [^15^O]H_2_O positron emission tomography (PET) perfusion imaging to assess presence and location of ischemia (Figure 3a). A large perfusion defect in the LAD territory with a hyperemic myocardial blood flow of 1.16 mL/min/g (below the ischemic threshold of 2.3 mL/min/g) was observed [2]. At the time of the second presentation, C-reactive protein (CRP) was 2 mg/L, compared to 5 mg/L during the initial presentation.

#### 2.2.4. Management

The patient was referred for ICA. The RCA and circumflex artery (Cx) had stenosis of intermediate severity that was functionally nonsignificant (FFR: 0.92 and 0.86, respectively) (Figure 2b (A,B)). Notably, the vulnerable plaque of the proximal LAD had progressed to a subtotal stenosis (Figure 2b (B,C)), for which a PCI was subsequently performed (Figure 2b (D)). Direct stenting was carried out using two DESs of 3.0 and 3.5-mm diameter, covering the lesion from the midsegment of the LAD to the ostial left main (LM). To ensure optimal stent expansion in the larger diameter of the LM, a proximal optimization technique (POT) was applied using a 5.0-mm noncompliant balloon inflated to 16 atm. The procedure resulted in an excellent angiographic outcome, with good stent apposition and preserved flow in both the diagonal (D) branch and the Cx (Figure 2b (D)). There was an interval of 4 months between the initial PCI and the second PCI.

#### 2.2.5. Follow-Up

After his second PCI, the patient was free of symptoms. A follow-up [^15^O]H_2_O PET scan performed 2 months later revealed normalization of perfusion (Figure 3b). No follow-up lipid profile was obtained after the second PCI.

## 3. Discussion

This report underscores the pathophysiological mechanisms underlying rapid coronary plaque progression and illustrates how multimodality imaging can aid in the detection and characterization of vulnerable plaques. A coronary plaque that is prone to cause an acute thrombotic event leading to myocardial infarction (MI) is considered “vulnerable” [3]. These vulnerable plaques are thought to rapidly progress in size prior to rupturing [3]. The present case demonstrates rapid progression of conservatively treated CCTA-defined vulnerable plaque despite lipid-lowering medication. However, the subsequent reappearance of symptoms occurred swiftly, limiting a comprehensive assessment of the initiated medication regimen's effectiveness. The accelerated timeline between interventions may have influenced observed outcomes, posing a challenge in fully discerning the medical treatment's impact on vulnerable plaque progression. In addition, potential traumatic influence on the LAD ostial plaque during the initial intervention was considered. However, it is crucial to note the absence of procedural complications, such as dissections or hematomas, indicating minimal trauma. The plaque's stability postintervention, demonstrated by a symptom-free period and a normal stress test result, suggests immediate destabilization was not induced. However, the subsequent recurrence of anginal symptoms within 1 month, followed by gradual worsening, suggests progressive plaque destabilization rather than an acute thrombotic event. This delayed onset of symptoms may be explained by positive vascular remodeling, where the plaque initially expands outward, maintaining luminal patency before eventually encroaching on the vessel lumen as the disease progresses [3]. This progression of clinical symptoms, with worsening over a relatively short period, aligns with the spectrum of acute coronary syndrome (ACS) [4].

Inflammation plays a pivotal role in atherosclerosis [5]. Low-density lipoproteins are taken up by macrophages which become foam cells and when clustered together form lipid-rich necrotic cores [5]. Neovascularization of the necrotic core is an important contributor to rapid plaque progression. The newly formed vessels are fragile and prone to cause intraplaque hemorrhage [3]. Inflammatory mediators not only drive this angiogenic process but also promote vascular permeability, facilitating the infiltration of immune cells and accelerating plaque destabilization. Furthermore, silent plaque rupture and subsequent healing possibly play an additional role in rapid plaque progression [6].

Patients with vulnerable plaques are at an elevated risk of subsequent MI [3, 7]. However, current guidelines do not recommend pre-emptive stenting of functionally irrelevant vulnerable plaques nor serial testing for assessment of vulnerable plaque progression. This seems justified, as most patients with a vulnerable plaque will not suffer an acute thrombotic event [8].

Nonetheless, the search for optimal treatment of nonflow-limiting vulnerable plaques is ongoing. Recently, the PROSPECT II trial [9] demonstrated that nonculprit vulnerable plaques are prone to cause future events. The PROSPECT ABSORB study [10] was embedded within the PROSPECT II trial and randomized 182 patients with nonflow-limiting vulnerable plaques to treatment with a bioresorbable vascular scaffold (BVS) or optimal medical therapy. Prophylactic treatment by BVS implantation was safe and significantly enlarged the coronary lumen compared to a conservative strategy [10]. The trial was, however, not powered to assess clinical outcomes. The recently published PREVENT trial [11] provided more definitive insights. In this multicenter, open-label, randomized controlled trial, 1606 patients with nonflow-limiting vulnerable plaques were assigned to PCI plus optimal medical therapy or optimal medical therapy alone. At 2 years, the primary outcome—composite of cardiac death, target vessel MI, target vessel revascularization, or unplanned hospitalization for angina—occurred in 0.4% of the PCI group compared to 3.4% of the medical therapy group (*p* = 0.0003), demonstrating a significant reduction in major adverse cardiac events with PCI [11]. This trial underscores the potential benefit of PCI in reducing adverse cardiac events in patients with nonflow-limiting vulnerable plaques. So, while most vulnerable plaques do not lead to acute thrombotic events, certain high-risk plaques may warrant closer monitoring or even pre-emptive intervention. The characteristics of the plaque in this case meet all three criteria of a vulnerable plaque (Figure 1), namely, low attenuation, positive remodeling, and the ring-like sign [12]. A more refined risk stratification could help determine which plaques may benefit from pre-emptive stenting. CCTA can play a crucial role in risk stratification by identifying which plaques may benefit from pre-emptive stenting and which can be managed conservatively [13].

However, emerging local therapies such as drug-coated balloons may offer a novel approach to stabilizing high-risk plaques without leaving a permanent implant. The DEBuT-LRP study [14] demonstrated that pre-emptive treatment of nonflow-limiting lipid-rich plaques with a paclitaxel-coated balloon was safe and led to a significant reduction in lipid burden at 9-month follow-up, suggesting a potential role in selected patients.

Besides invasive treatment, novel medical treatments might prevent events in patients with a vulnerable plaque. The LoDoCO2 trial [15] demonstrated that an anti-inflammatory treatment in patients with chronic CAD led to a significant reduction of events compared to placebo. Furthermore, Budoff et al. [16] showed icosapent ethyl treatment to significantly reduce low-attenuation plaque volume compared to placebo. Although speculative, these types of treatments might be more beneficial among patients with vulnerable plaques by facilitating plaque stabilization and thereby possibly preventing rapid plaque progression.

### 3.1. Conclusion

CCTA-derived vulnerable plaques without significant lumen narrowing might progress in a relatively short period of time into flow-limiting stenoses with critical lumen narrowing.

## Figures and Tables

**Table 1:** Cardiovascular risk factors and medication at first presentation.

**Cardiovascular risk factors**	

BMI	26 kg/m^2^
Blood pressure	133/65 mmHg
LDL	2.2 mmol/L
HDL	1.3 mmol/L
Smoking	Stopped smoking at the age of 25 after 11 pack-years
Alcohol	None
Family medical history	Father and brother experiencing myocardial infarction at an age around 40 years

**Medication at first presentation**	

Omeprazole capsule extended release 20 mg orally, 2 tablets once daily	
Fluticasone nasal drops 1 mg/mL, 1 drop per nostril twice a day	
Verapamil tablet 120 mg orally, as needed once daily	

Abbreviations: BMI, body mass index; HDL, high-density lipoprotein; LDL, low-density lipoprotein.

**Table 2:** Medical management at first presentation.

Atorvastatin tablet 10 mg orally, 2 tablets once daily
Carbasalate calcium powder 100 mg orally, 1 powder once daily
Isosorbide mononitrate extended-release tablet 60 mg orally, 0.5 or 2 tablets once daily
Metoprolol succinate tablet 50 mg orally, 1 tablet once daily
Nitroglycerin sublingual spray 0.4 mg per dose, administer sublingually as needed
Verapamil tablet 120 mg orally, as needed

## Data Availability

No datasets were generated or analyzed during the current study.
